# The Hydroalcoholic Extract of *Uncaria tomentosa* (Cat's Claw) Inhibits the Infection of Severe Acute Respiratory Syndrome Coronavirus 2 (SARS-CoV-2) *In Vitro*

**DOI:** 10.1155/2021/6679761

**Published:** 2021-02-24

**Authors:** Andres F. Yepes-Perez, Oscar Herrera-Calderón, Cristian A. Oliveros, Lizdany Flórez-Álvarez, María I. Zapata-Cardona, Lina Yepes, Wbeimar Aguilar-Jimenez, María T. Rugeles, Wildeman Zapata

**Affiliations:** ^1^Chemistry of Colombian Plants, Institute of Chemistry, Faculty of Exact and Natural Sciences, University of Antioquia-UdeA, Calle 70 No. 52-21, A.A 1226, Medellin, Colombia; ^2^Academic Department of Pharmacology, Bromatology and Toxicology, Faculty of Pharmacy and Biochemistry, Universidad Nacional Mayor de San Marcos, Jr Puno 1002, Lima 15001, Peru; ^3^Biomolecules Research Center, CIBIMOL, Universidad Industrial de Santander, UIS, Carrera 27 Calle 9, Bucaramanga, Colombia; ^4^Grupo Inmunovirología, Facultad de Medicina, Universidad de Antioquia UdeA, Medellín, Colombia; ^5^Grupo Infettare, Facultad de Medicina, Universidad Cooperativa de Colombia, Medellín, Colombia

## Abstract

The coronavirus disease 2019 (COVID-19) has become a serious problem for public health since it was identified in the province of Wuhan (China) and spread around the world producing high mortality rates and economic losses. Nowadays, the WHO recognizes traditional, complementary, and alternative medicine for treating COVID-19 symptoms. Therefore, we investigated the antiviral potential of the hydroalcoholic extract of *Uncaria tomentosa* stem bark from Peru against SARS-CoV-2 *in vitro*. The antiviral activity of *U. tomentosa* against SARS-CoV-2 *in vitro* was assessed in Vero E6 cells using cytopathic effect (CPE) and plaque reduction assay. After 48 h of treatment, *U. tomentosa* showed an inhibition of 92.7% of SARS-CoV-2 at 25.0 *μ*g/mL (*p* < 0.0001) by plaque reduction assay on Vero E6 cells. In addition, *U. tomentosa* induced a reduction of 98.6% (*p*=0.02) and 92.7% (*p*=0.03) in the CPE caused by SARS-CoV-2 on Vero E6 cells at 25 *μ*g/mL and 12.5 *μ*g/mL, respectively. The EC50 calculated for the *U. tomentosa* extract by plaque reduction assay was 6.6 *μ*g/mL (4.89–8.85 *μ*g/mL) for a selectivity index of 4.1. The EC50 calculated for the *U. tomentosa* extract by TCID50 assay was 2.57 *μ*g/mL (1.05–3.75 *μ*g/mL) for a selectivity index of 10.54. These results showed that U. tomentosa, known as cat's claw, has an antiviral effect against SARS-CoV-2, which was observed as a reduction in the viral titer and CPE after 48 h of treatment on Vero E6 cells. Therefore, we hypothesized that *U. tomentosa* stem bark could be promising in the development of new therapeutic strategies against SARS-CoV-2.

## 1. Introduction

The severe acute respiratory syndrome coronavirus 2 (SARS-CoV-2) has caused serious public health problems since it was identified in Wuhan (China) in late 2019 [1]. The World Health Organization (WHO) declared coronavirus disease 2019 (COVID-19) a pandemic on March 11, 2020 [2]. According to the latest report of the WHO, there have been 88,383,771 confirmed cases of COVID-19, including 1,919,126 deaths, as of 10 January 2021 [3]. When the novel coronavirus (SARS-CoV-2) arrived in Latin America, Brazil was the first South American country to declare a patient with COVID-19 whereas Venezuela and Uruguay were the ultimate nations to confirm their patient zero, considering the pandemic epicenter after Europe [4]. Even though some vaccines have already been approved only with phase 3 results, currently, there is no preventive treatment or antiviral drug available against SARS-CoV-2 [5].

Nowadays, the World Health Organization (WHO) recognizes that traditional, complementary, and alternative medicine has many benefits [6]. Several candidates with possible antiviral effects have been explored from medicinal plants in the preclinical phase. *Uncaria tomentosa* (Willd.) DC. (*U. tomentosa*) belongs to the Rubiaceae family, which is also known as cat's claw and contains more than 50 phytochemicals [7]. Oxindole alkaloids (pentacyclic oxindole alkaloids (POA) and tetracyclic oxindole alkaloids (TOA)) have been recognized as a fingerprint of this species in some pharmacopeias, and several pharmacological activities are linked to this kind of alkaloids [8, 9]. It has been demonstrated that *U. tomentosa* exerts an antiviral effect on human monocytes infected with dengue virus 2 (DENV-2) [10] and herpes simplex virus type 1 (HSV-1) [11]. In our previous studies *in silico*, *U. tomentosa'*s components inhibited the SARS-CoV-2 enzyme 3CLpro and disrupted the interface of the receptor-binding domain of angiotensin-converting enzyme 2 (RBD-ACE-2) as well as the SARS-CoV-2 spike glycoprotein [12, 13]. Additionally, bioactivities such as anti-inflammatory [14], antiplatelet [15], and immunomodulatory [16] were reported in the literature. Furthermore, other components isolated from the stem bark such as quinovic acids, polyphenols (flavonoids, proanthocyanidins, and tannins), triterpenes, glycosides, and saponins were identified by instrumental methods [9, 17–20].

The evaluation of natural compounds to inhibit SARS-CoV-2 in preclinical studies might lead to discovering new antiviral drugs and to a better understanding of the viral life cycle [21]. Several cell lines such as human airway epithelial cells, Vero E6 cells, Caco-2 cells, Calu-3 cells, HEK293T cells, and Huh7 cells are considered the best models *in vitro* to determine the antiviral activity against SARS-CoV-2 [22]. Vero E6 cells highly express the ACE-2 receptor; they produce a high titer of viral particles and do not produce interferon [22]. Therefore, *in vitro* test in this cell line constitutes the first step at the beginning of the antiviral studies.

Although the pathophysiology of COVID-19 is not completely understood, a severe inflammatory process has been associated with the severity and progression of the disease [23]. Therefore, the immune activation so far described during the course of the infection as well as the pulmonary injury could be ameliorated by *U. tomentosa* linked to its traditional use as an anti-inflammatory in the folk medicine from South America for years [24].

Based on its antiviral activity on other ARN viruses and our *in silico* findings against SARS-CoV-2, we assayed the hydroalcoholic extract of *U. tomentosa* stem bark from Peru as a potential antiviral agent *in vitro* against this severe acute respiratory syndrome coronavirus 2.

## 2. Material and Method

### 2.1. Plant Material

*U. tomentosa* (cat's claw) used in this investigation is dispensed to patients of the Medicine Complementary Service of EsSalud (Social Health Insurance) in Peru for inflammatory disorders. The raw material (stem bark) of *U. tomentosa* was sourced from the Pharmacy Office of EsSalud in Ica, Peru. Next, the sample was transported to the Faculty of Medicine of the Universidad Nacional Mayor de San Marcos (UNMSM, Lima, Peru), in order to obtain the hydroalcoholic extract.

### 2.2. Obtaining Extract from Plant Material

One hundred grams of the raw plant material (stem bark) of *U. tomentosa* was powdered and extracted with 700 ml of 70% ethanol at room temperature for 7 days. Then, the extract was evaporated by using rotary evaporation to obtain a desiccated extract, which was stored at 4°C until further use.

### 2.3. Identification of the *U. tomentosa* Stem Bark Constituents by LC/MS (UHPLC-ESI + -HRMS-Orbitrap)

The identification of the main phytochemicals present within the hydroalcoholic extract of *U. tomentosa* was carried out on an LC Dionex UltiMate 3000 (Thermo Scientific, Germering, Germany) equipped with a degassing unit, a gradient binary pump, an autosampler with 120-vial well-plate trays, and a thermostatically controlled column compartment. The autosampler was held at 10 °C, and the column compartment was maintained at 40 °C. Chromatographic separation was performed on a Hypersil GOLD aQ column (Thermo Scientific, Sunnyvale, CA, USA; 100 mm × 2.1 mm id, 1.9 *μ*m particle size) with an LC guard-column Accucore aQ Defender cartridge (Thermo Scientific, San Diego, CA, USA; 10 × 2.1 mm id, 2.6 *μ*m particle size). The flow rate of the mobile phase containing ammonium formate (FA)/water (A) and FA/acetonitrile (B) was 300 *μ*L/min. The initial gradient condition was 100% A, changed linearly to 100% B in 8 min, maintained for 4 min, returned to 100% A in 1 min, and maintained for 3 min. The injection volume was 1 *μ*L. The LC was connected to an Exactive Plus Orbitrap mass spectrometer (Thermo Scientific, Bremen, Germany) with a heated electrospray ionization (HESI-II) source operated in the positive ion mode. The Vspray was evaluated at 1.5, 2.5, 3.5, and 4.5 kV. The nebulizer temperature was set at 350°C; the capillary temperature was 320°C; sheath gas and auxiliary gas (N_2_) were adjusted to 40 and 10 arbitrary units, respectively. Nitrogen (>99%) was obtained from a generator (NM32LA, Peak Scientific, Scotland, UK). During the full scan MS, the Orbitrap-MS mass resolution was set at 70000 (full-width-at-half-maximum, at *m*/*z* 200, RFWHM) with an automatic gain control (AGC) target of 3 × 106, a C-trap maximum injection time of 200 ms, and a scan range of *m*/*z* of 100–1000. The ions injected to the HCD cell via the C-trap were fragmented with stepped normalized collision energies of 20, 30, 40, and 50 eV. The mass spectra were recorded in the AIF (all-ion fragmentation) mode for each collision energy at an RFWHM of 35000, an AGC target of 3 × 106, a C-trap injection time of 50 ms, and a mass range of *m*/*z* of 80–1000. Full instrument calibration was performed every week using a Pierce LTQ Velos ESI Positive Ion Calibration Solution (Thermo Scientific, Rockford, IL, USA). The data obtained were analyzed using Thermo Xcalibur 3.1 software (Thermo Scientific, San Jose, CA, USA).

### 2.4. Preparation of Stock Solution of *U. tomentosa* Extract

One milligram of *U. tomentosa* hydroalcoholic extract was suspended in 1 mL of DMSO. The solution was maintained at room temperature, protected from light until use. To prepare a working solution, the stock was diluted to 50 mg/mL in DMEM supplemented with 2% fetal bovine serum (FBS) (5% final concentration DMSO).

### 2.5. Cell Lines and Virus

Vero E6 epithelial cell line from *Cercopithecus aethiops* kidney was donated by Instituto Nacional de Salud (INS) (Bogotá, Colombia). Cells were maintained in Dulbecco's modified Eagle's medium (DMEM) supplemented with 2% FBS and 1% penicillin-streptomycin. Cultures were maintained at 37°C, with 5% CO_2_. Infections were done with a viral stock produced from a Colombian isolate of SARS-CoV-2 (hCoV-19/Colombia/ANT-UdeA-200325-01/2020) [25].

### 2.6. Cell Viability Assays

The viability of Vero E6 cells in the presence of the *U. tomentosa* extract was evaluated using an MTT (4,5-dimethylthiazol-2-yl)-2,5-diphenyl tetrazolium bromide) assay. Briefly, Vero E6 cells were seeded at a cell density of 1.0 × 10^4^ cells/well in 96-well plates and incubated for 24 h at 37°C in a humidified 5% CO_2_ atmosphere. Then, 100 *μ*L of serial dilutions (1 : 2) of the *U. tomentosa* extract ranging from 3.1 to 50 *μ*g/mL was added to each well and incubated for 48 h at 37°C with 5% CO_2_. After incubation, the supernatants were removed, cells were washed twice with phosphate-buffered saline (PBS) (Lonza, Rockland, ME, USA), and 30 *μ*L of the MTT reagent (Sigma-Aldrich) (2 mg/mL) was added. The plates were incubated for 2 hours at 37°C with 5% CO_2_, protected from light. Then, formazan crystals were dissolved by adding 100 *μ*L of pure DMSO to each well. Plates were read using a Multiskan GO spectrophotometer (Thermo) at 570 nm. The average absorbance of cells without treatment was considered as 100% of viability. Based on this control, the cell viability of each treated well was calculated. The treatment concentration with 50% cytotoxicity (the 50% cytotoxic concentration, CC50) was obtained by performing nonlinear regression followed by the construction of a concentration-response curve (GraphPad Prism). For the MTT assay, 2 independent experiments with four replicates of each experiment were performed (*n* = 8).

### 2.7. Antiviral Assay

The antiviral activity of the *U. tomentosa* extract against SARS-CoV-2 was evaluated with a pre-post strategy where the treatment was added before and after the infection. Briefly, Vero E6 cells were seeded at a density of 1.0  ×  10^4^ cells/well in 96-well plates and incubated for 24 h at 37°C with 5% CO_2_. After incubation, 50 *μ*L of double dilutions of cat's claw (3.1–25 *μ*g/mL) was added to the cell monolayers for 1 h at 37°C with 5% CO_2_. Then, the treatment was removed, and cells were infected with SARS-CoV-2 stock at a multiplicity of infection (MOI) of 0.01 in 50 *μ*L of DMEM supplemented with 2% FBS. The inoculum was removed 1 hour postinfection (h.p.i), replaced by 170 *μ*L of cat's claw dilutions, and incubated for 48 h at 37°C with 5% CO_2_. Then, cell culture supernatants were harvested and stored at −80 °C for virus titration by plaque assay and TCID50 assay. The supernatant of infected cells without treatment was used as infection control. Chloroquine (CQ) at 50 *μ*M was used as a positive control for antiviral activity; 2 independent experiments with 3 replicates of each experiment were performed (*n* = 6).

#### 2.7.1. Plaque Assay for SARS-CoV-2 Quantification

The capacity of the *U. tomentosa* extract to decrease the PFU/mL of SARS-CoV-2 was evaluated by plaque assay on Vero E6 cells. Briefly, 1.0 × 10^5^ Vero E6 cells per well were seeded in 24-well plates for 24 h at 37°C with 5% CO_2_. Tenfold serial dilutions of the supernatants obtained from the antiviral assay (200 *μ*L per well) were added by duplicate on cell monolayers. After incubation for 1 h at 37°C with 5% CO_2_, the viral inoculum was removed and 1 mL of semisolid medium (1.5% carboxymethyl cellulose in DMEM 1X with 2% FBS and 1% penicillin-streptomycin) was added to each well. Cells were incubated for 5 days at 37°C with 5% CO_2_. Then, cells were washed twice with PBS. Then, cells were fixed and stained with 500 *μ*L of 4% formaldehyde/1% crystal violet solution for 30 minutes and washed with PBS. Plaques obtained from each condition were counted. The reduction in the viral titer after treatment with each concentration of the *U. tomentosa* extract compared to the infection control is expressed as inhibition percentage. Two independent experiments with two replicates of each experiment were performed (*n* = 4).

#### 2.7.2. TCID50 Assay for SARS-CoV-2 Quantification

The capacity of the *U. tomentosa* extract to diminish the CPE caused by SARS-CoV-2 on Vero E6 cells was evaluated by TCID50 assay. Briefly, 1.2 × 10^4^ Vero E6 cells per well were seeded in 96-well plates for 24 h at 37°C with 5% CO_2_. Tenfold serial dilutions of the supernatants obtained from the antiviral assay (50 *μ*L per well) were added by quadruplicate on cell monolayers. After 1 h incubation, at 37°C with 5% CO_2_, the viral inoculum was removed and replaced by 170 *μ*L of DMEM supplemented with 2% FBS. Cells were incubated for 5 days at 37°C with 5% CO_2_. Then, cells were washed twice with PBS and then fixed and stained with 100 *μ*L/well of 4% formaldehyde/1% crystal violet solution for 30 minutes. Cell monolayers were washed with PBS. The number of wells positive for CPE was determined for each dilution (CPE is considered positive when more than 30% of cell monolayer is compromised).

The viral titer of TCID50/mL was calculated based on the Spearman–Käerber method. The reduction of viral titer after treatment with each concentration of the *U. tomentosa* extract compared to infection control is expressed as inhibition percentage. A control of cells without infection and treatment was included. Two independent experiments with two replicates of each experiment were performed (*n* = 4).

### 2.8. Statistical Analysis

The median inhibitory concentration (IC50) values represent the concentration of the *U. tomentosa* extract that reduces virus particle production by 50%. The CC50 values represent the cat's claw solution concentration that causes 50% cytotoxicity. The corresponding dose-response curves were fitted by nonlinear regression analysis using a sigmoidal model. The calculated selectivity index (SI) represents the ratio of CC50 to IC50. All data were analyzed with GraphPad Prism (La Jolla, CA, USA), and data are presented as mean ± SEM. Statistical differences were evaluated via Student's *t*-test or Mann–Whitney *U* test; a value of *p* ≤ 0.05 was considered significant, with ^*∗*^*p* ≤ 0.05, ^*∗∗*^*p* ≤ 0.01, and ^*∗∗∗*^*p* ≤ 0.001.

## 3. Results

### 3.1. Identification of Components in the Hydroalcoholic Extract of *U. tomentosa* by LC/MS (UHPLC-ESI + -HRMS-Orbitrap)

Different constituents in the *U. tomentosa* stem bark such as spirooxindole alkaloids, indole glycoside alkaloids, quinovic acid glycosides, and proanthocyanidins were identified by LC-MS analysis (Table 1 and Supplementary materialsS1–S6). The LC-MS data provided information on spirooxindole alkaloids as a broad peak that appeared at a retention time (*t*_*R*_) of 4.82 min and showed an (M + H) + ion at *m*/*z* 369.18018 that are characteristics for speciophylline, isopteropodine, isomitraphylline, uncarine F, mitraphylline, and pteropodine. Furthermore, two peaks at 4.99 and 5.18 min, respectively, showed the [M+H] + ion at *m*/*z* 385.21127 that were identified as those isomeric spirooxindole-related alkaloids rhynchophylline and isorynchophylline. On the other side, a molecular ion peak (*M* + *H*) +  of 547.22992 *m*/*z*, which eluted at 4.03 min, provided the identity of the indole glycoside alkaloid 3-dihydrocadambine. As expected, LC/MS phytochemical analysis showed that the hydroalcoholic extract of *U. tomentosa* was comprised predominantly of five proanthocyanidins (PAs), including proanthocyanidin C1, epiafzelechin-4*β*-8, proanthocyanidin B2, epicatechin, and chlorogenic acid, which eluted at 3.76–4.25 min. Finally, LC-MS data along with ESI mass spectra gave characteristic protonated quasimolecular ions of isomeric quinovic acid glycosides ([M + H] +  ion at *m*/*z* 957.50458). In sum, LC/MS allowed the identification of known components in the hydroalcoholic extract of *U. tomentosa* used, such as alkaloids, quinovic acid glycosides, and proanthocyanidins (PAs), which play important roles in the biological activities of this medicinal herb and are considered as a fingerprint for quality control that ensures fitness for therapeutic uses.

### 3.2. The  Cell Viability Assay on Vero E6 Cells in the Presence of the *U. tomentosa* Extract

**Figure 1:**
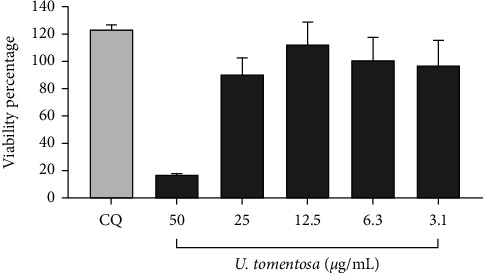
Viability of Vero E6 cells in the presence of the *U. tomentosa* extract. The figure represents the viability percentage of Vero E6 cells after 48 h of treatment with *U. tomentosa* (3.1 to 50.0 *μ*g/mL). The viability percentages of treated cells were calculated based on the average absorbance control of cells without treatment. Chloroquine (CQ) was used as an inhibition control of the antiviral strategy. Bars represent mean values ± SEM (2 independent experiments with four replicates of each experiment were performed, *n* = 8).

The viability of Vero E6 cells in the presence of *U. tomentosa* was higher than 90.0% at concentrations of 25.0 *μ*g/mL or lower, after 48 h of incubation (Figure 1). Cell viability at 50.0 *μ*g/mL was 17.3%; for this reason, this concentration was not included in the antiviral assay. The CC50 calculated for *U. tomentosa* was 27.1 *μ*g/mL. Chloroquine at 50 *μ*M (positive control of inhibition) did not affect the viability of Vero E6 cells (Figure 1).

### 3.3. The *U. tomentosa* Extract Inhibited the Number of Infectious Viral Particles of SARS-CoV-2

**Figure 2:**
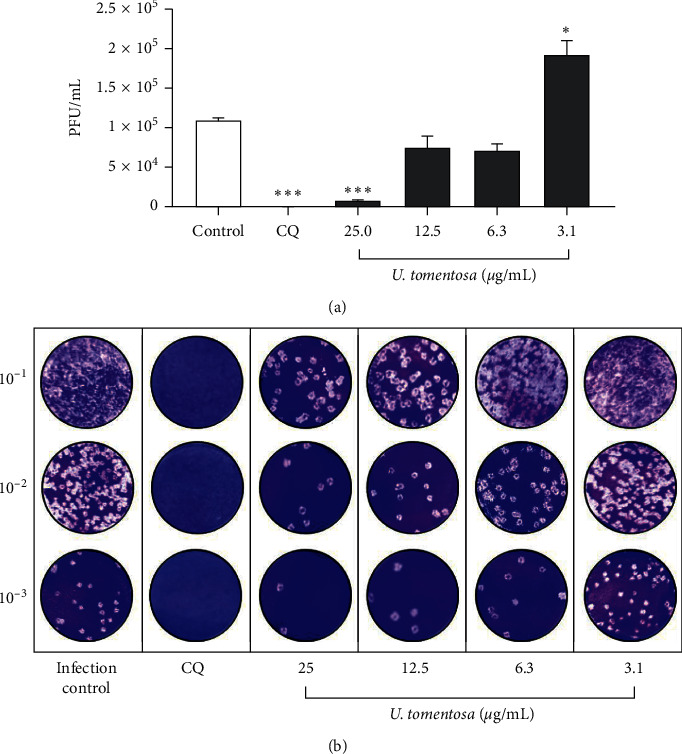
Antiviral activity *in vitro* of the *U. tomentosa* extract against SARS-CoV-2 by plaque assay. (a) The figure represents the viral titer (PFU/mL) of supernatants harvested after the treatment with the *U. tomentosa* extract quantified by plaque assay (*n* = 4). Chloroquine (CQ) was used as an inhibition positive control of the antiviral strategy. ^*∗*^*p* ≤ 0.05, ^*∗∗*^*p* ≤ 0.01, and ^*∗∗∗*^*p* ≤ 0.001 (b) Representative plaques of the antiviral evaluation of the *U. tomentosa* extract against SARS-CoV-2 on Vero E6 cells.

An inhibition of 92.7% of SARS-CoV-2 was obtained after the treatment with *U. tomentosa* at 25.0 *μ*g/mL (*p* < 0.0001) by plaque reduction assay (Figure 2). The *U. tomentosa* extract also showed an inhibition of 31.4% and 34.9% of SARS-CoV-2 at 12.5 and 6.3 *μ*g/mL, respectively (Figure 2). An increase of 76.0% of PFU/mL of SARS-CoV-2 was obtained after the treatment with the *U. tomentosa* extract at 3.1 *μ*g/mL (*p*=0.02) (Figure 2). The EC50 calculated for the extract by plaque assay was 6.6 *μ*g/mL (4.89–8.85 *μ*g/mL) for a selectivity index of 4.1. Chloroquine (inhibition positive control) showed an inhibition of 100% of SARS-CoV-2 at 50 *μ*M (*p* < 0.0001) (Figure 2).

### 3.4. The *U. tomentosa* Extract Reduced the CPE of SARS-CoV-2

**Figure 3:**
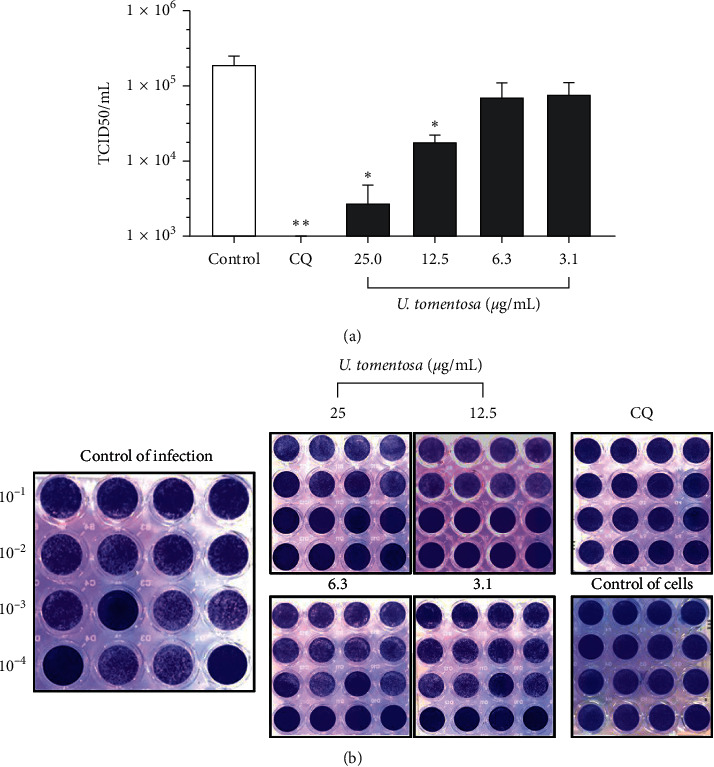
Antiviral activity *in vitro* of the *U. tomentosa* extract against SARS-CoV-2 by TCID50 assay. (a) The figure represents the viral titer (TCID50/mL) quantified by TCID50 assay on supernatants harvested from the treatment with the *U. tomentosa* extract (*n* = 4). Chloroquine (CQ) was used as an inhibition positive control of the antiviral strategy. ^*∗*^*p* ≤ 0.05 and ^*∗∗*^*p* ≤ 0.01. (b) Representative images of the antiviral evaluation of the *U. tomentosa* extract against SARS-CoV-2 on Vero E6 cells by TCID50 assay revealed by crystal violet.

The *U. tomentosa* extract induced a reduction of 98.6% (*p*=0.02), 92.7% (*p*=0.03), 63.2%, and 60.4% in the CPE caused by SARS-CoV-2 on Vero E6 cells at 25, 12.5, 6.3, and 3.1 *μ*g/mL, respectively (Figure 3). The EC50 calculated for the *U. tomentosa* extract by TCID50 assay was 2.57 *μ*g/mL (1.05–3.75 *μ*g/mL) for a selectivity index of 10.54. Chloroquine showed an inhibition of 100% in the CPE of SARS-CoV-2 on Vero E6 cells at 50 *μ*M (*p*=0.008) (Figure 3).

## 4. Discussion

In South America, the second wave of novel coronaviruses might be more aggressive, increasing the mortality rate and new cases [26]. Medical trials are underway to determine the efficacy of several vaccines against SARS-CoV-2 [27]. Otherwise, herbal medicines could become a promising option to tackle the ongoing pandemic caused by COVID‐19 [28]. Some plant extracts and phytochemicals were modeled over numerous targets of SARS-CoV-2 by using *in silico* studies, which is the first step in the discovery of new drugs [29]. In China, the use of herbal formulas has been included in the protocol of primary attention in COVID-19 and medical trials were carried out, and promising results to ameliorate the symptoms were reported [30].

Our previous study of *U. tomentosa* (cat's claw) on this novel coronavirus using *in silico* analysis showed that two possible mechanisms could be involved in the *in vitro* antiviral activity against SARS-CoV-2. These findings revealed that 3CLpro, an essential enzyme for viral replication [31], showed key molecular interactions with speciophylline, cadambine, and proanthocyanidin B2, with high binding affinities ranging from −8.1 to −9.2 kcal/mol. [12]. On the other hand, phytochemicals of *U. tomentosa* such as proanthocyanidin C1, QAG-2, uncarine F, 3-isodihydrocadambine, and uncaric acid (docking scores: −8.6, −8.2, −7.1, −7.6, and −7.0 kcal/mol, respectively) showed high binding affinity for the interface of the RBD-ACE-2. In addition, 3-dihydrocadambine, proanthocyanidin B4, proanthocyanidin B2, and proanthocyanidin C1 (−7.1, −7.2, −7.2, and −7.0 kcal/mol, respectively) had the highest binding score on SARS-CoV-2 spike glycoprotein [13]. Since Vero E6 cells are commonly used to replicate SARS-CoV-2 due to the high expression level of the ACE-2 receptor and lack the ability to produce interferon [32], phytochemicals are the appropriate substrate to explore the antiviral activity of phytochemicals targeting the receptor binding as well as the SARS-CoV-2 main protease, which is a high-profile antiviral drug target, and several compounds have been discovered as main protease inhibitors [33, 34].

Mechanisms of the antiviral activity of the hydroalcoholic extract of *U. tomentosa*, on other viruses like Dengue (DEN-2), have been elucidated; alkaloids (pentacyclic alkaloids) from *U. tomentosa* induced apoptosis of infected cells and reduced inflammatory mediators such as TNF-*α* and IFN-*α* with similar effects to dexamethasone [10]. The quinovic acids (33.1–60 *μ*g/mL) inhibited the vesicular stomatitis virus (VSV) [35], and the total extract at concentrations less than 15.75 *μ*g/mL inhibited the herpes simplex virus (HSV-1) replication when added to Vero cells at the same time compared to the virus [11].

Here, we demonstrated that *U. tomentosa* also has an antiviral activity *in vitro* against the SARS-CoV-2 by inhibiting the release of infectious particles and reducing the cytopathic effect on Vero E6 cells. The EC50 was calculated at 6.6 *μ*g/mL (95% CI: 4.89–8.85 *μ*g/mL) by plaque assay and at 2.57 *μ*g/mL (95% CI: 1.05–3.75 *μ*g/mL) by TCID50 assay, whilst the CC50 was 27.1 *μ*g/mL. In other medicinal plants assayed against SARS-CoV-2, similar antiviral activity was shown; in particular, Echinaforce® (an *Echinacea purpurea* preparation) exhibited an antiviral activity at 50 *μ*g/mL [36]. Liu Shen capsule, a traditional Chinese medicine, inhibited the SARS-CoV-2 replication with an EC50 value of 0.6024 *μ*g/mL and CC50 of 4.930 *μ*g/mL [37]. Likewise, phillyrin (KD-1), a representative constituent of *Forsythia suspensa* (Thunb.), presented an EC50 at 63.90 *μ*g/mL and CC50 of 1959 *μ*g/mL [38]. Sulfated polysaccharides named RPI-27 and heparin inhibited SARS-CoV-2 *in vitro* with an EC50 of 8.3 ± 4.6 *μ*g/mL and 36 ± 14 *μ*g/mL, respectively [39]. In our study, selectivity indices of 4.1 and 10.5 were obtained by plaque assay and TCID50, respectively. According to a previous report [40], these results were classified as low selectivity (SI ≥ 2.0 and < 5) and high selectivity (SI ≥ 10), respectively. In spite of SI having a low value, theoretically having a higher value would be more effective and safer during *in vivo* treatment for a given viral infection. However, there is no evidence of severe toxicity of *U. tomentosa*, and traditionally, its popular use in the form of maceration or decoction is safe [41].

The lower concentration used of the *U. tomentosa* extract (3.1 *μ*g/mL) caused a significant increase in the number of infectious viral particles compared to the infection control (Figure 2). This result could be due to compounds present in the extract that at this concentration promote an increase in cell proliferation or regulation of metabolic pathways that regulate the expression of viral receptors or synthesis of proteins necessary for the viral replicative cycle [42, 43]. These findings demonstrate the importance of evaluating and identifying the compounds present in *U. tomentosa* with antiviral effect against SARS-CoV-2 and selecting the proper concentration for use.

There is enough evidence that *U. tomentosa* could ameliorate a wide array of symptoms associated with COVID-19, like the severe inflammation characterized by a cytokine storm [24] causing endothelial dysfunction. According to the antiviral activity of *U. tomentosa* against SARS-CoV-2, several biochemical mechanisms could be involved in each phase of the viral life cycle. As previously reported in our *in silico* studies, *U. tomentosa* could interfere with viral entrance into host cells [12], affecting viral replication [13]. Furthermore, ACE-2 receptors, which are expressed in Vero E6 cells, could also be blocked by the phytochemicals of *U. tomentosa* during the entrance of SARS-CoV-2 into the host cells, and the aforementioned studies backed up our hypothesis [13].

Besides, it might control the hyperinflammation, via inhibition of IL-1*α*, IL-1*β*, IL-17, and TNF-*α* [44], reduce oxidative stress [45], and protect the endothelial barrier, via inhibition of IL-8, which is linked to the induction of permeability [46]. It also has antithrombotic potential via antiplatelet mechanism and by thrombin inhibition [15]. Furthermore, *U. tomentosa* modulates the immune system by extending lymphocyte survival via an antiapoptotic mechanism [47]. It is known that the 3*α* protein of severe acute respiratory syndrome-associated coronavirus induces apoptosis in Vero E6 cells [48]; therefore, the phytochemicals found in the hydroalcoholic extract could inhibit this process and protect against the inflammatory cascade. Interestingly, *U. tomentosa* bark extract reduced the lung inflammation produced by ozone in mice [49].

Based on our results, *U. tomentosa* is a promising medicinal herb to combat COVID-19, but it is necessary to continue with animal models followed by clinical trials to validate our results in the context of COVID-19 patients. This study is the first approach to evaluate the potential use of *U. tomentosa* against SARS-CoV-2; we have to explore specific mechanisms of inhibition and propose the main molecules involved with the antiviral activity. As shown in our phytochemical analysis, the presence of chemical groups determined by LC/MS (UHPLC-ESI + -HRMS-Orbitrap), such as spirooxindole alkaloids, indole glycoside alkaloids, quinovic acid glycosides, and proanthocyanidins, suggests that they could be responsible for the described activity. Here, the mechanisms discussed about the hydroalcoholic extract of *U. tomentosa* are only inferred under the mechanisms evaluated in other RNA viruses reported in the literature and also our previous *in silico* studies on SARS-CoV-2.

In regard to the antiviral activity of *U. tomentosa*, the EC50 was calculated at 6.6 *μ*g/mL, which is an indicator of a promising activity as an extract, but it cannot be taken as a reference value to reach plasma concentration because *U. tomentosa* extract presented several phytochemicals, which were not quantified and individually tested. Since cat's claw has been used in clinic for other diseases, there are no clinical studies carried out and reported pharmacokinetic data. However, in mice, the administration of 5 mg/Kg per oral of six Uncaria alkaloids presented a bioavailability ranging between 27.3% and 68.9% and with a maximum plasma concentration (*C*_max_) between 305.3 ± 68.8 ng/mL and 524.5 ± 124.5 ng/mL [50].

Additionally, the recommended dose of *U. tomentosa* is one gram given two to three times daily [51]. A standardized extract consisting of less than 0.5% oxindole alkaloids and 8% to 10% carboxy alkyl esters has been used at doses of 250 to 300 mg in clinical studies [52]. In humans, no toxic symptoms were reported with a usual administration of 350 mg/day for 6 weeks [53, 54] and 300 mg dry extract daily for 12 weeks [55]. Traditional uses such as tinctures, decoctions, capsules, extracts, and teas are prepared and, in a decoction, up to 20 g of raw bark per liter of water has been used; although this information is based on traditional practices, this equates to 4 mg oxindole alkaloids [56]. Thus, we hypothesized that the antiviral activity on SARS-CoV-2 is attributed to the whole extract synergized by all its phytochemicals acting by different mechanisms discussed above.

## 5. Conclusion

*U. tomentosa* has been widely used as an anti-inflammatory and immunomodulatory agent. Previous studies have shown that *U. tomentosa* has a broad spectrum of effects on several RNA viruses. In this study, we demonstrated that hydroalcoholic extract of *U. tomentosa* stem bark inhibited the release of SARS-CoV-2 infectious particles and reduced the cytopathic effect caused by the virus on Vero E6 cell line, underlying the importance of continuing this investigation with specific *in vitro* assays, followed by studies in animal models, and finally validating its use in clinical trials. Our investigation shows for the first time the antiviral effect of *U. tomentosa* on this novel coronavirus (SARS-CoV-2).

## Figures and Tables

**Table 1:** LC/MS phytochemical analysis of the hydroalcoholic extract of *U. tomentosa*.

Peak	Compounds of cat's claw	*t*_*R*_ (min)	*m*/*z* (M + H)^+^	Molecular formula	Chemical structure
Spirooxindole alkaloids					
1	Speciophylline	4.82	369.18018	C_21_H_24_N_2_O_4_	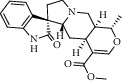
1	Isopteropodine	4.82	369.18018	C_21_H_24_N_2_O_4_	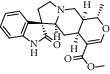
1	Isomitraphylline	4.82	369.18018	C_21_H_24_N_2_O_4_	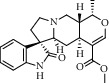
1	Uncarine F	4.82	369.18018	C_21_H_24_N_2_O_4_	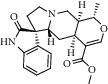
1	Mitraphylline	4.82	369.18018	C_21_H_24_N_2_O_4_	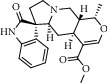
1	Pteropodine	4.82	369.18018	C_21_H_24_N_2_O_4_	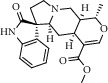
2	Rhynchophylline	4.99	385.21127	C_22_H_28_N_2_O_4_	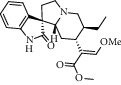
3	Isorynchophylline	5.19	385.21140	C_22_H_28_N_2_O_4_	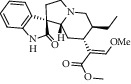

Indole glycoside alkaloids					
3	3-Dihydrocadambine	4.03	547.22992	C_27_H_34_N_2_O_10_	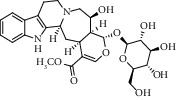

Quinovic acid glycosides					
4	QAG-1	4.84	957.50458	C_48_H_77_O_19_	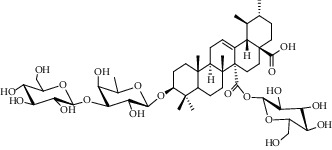
4	QAG-2	4.84	957.50458	C_48_H_77_O_19_	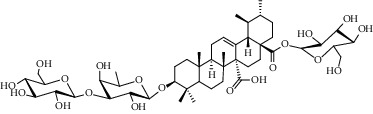

Proanthocyanidins					
5	Proanthocyanidin C1	4.17	867.21191	C_45_H_38_O_18_	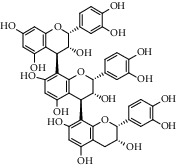
6	Epiafzelechin-4*β*-8	4.25	563.15460	C_30_H_26_O_11_	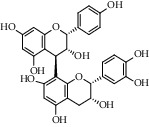
7	Proanthocyanidin B2	4.01	579.15002	C_30_H_26_O_12_	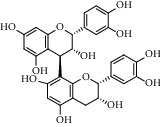
7	Proanthocyanidin B4	4.01	579.15002	C_30_H_26_O_12_	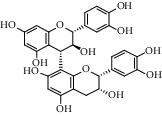
8	Epicatechin	4.15	291.08588	C_15_H_14_O_6_	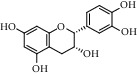
9	Chlorogenic acid	3.95	355.10220	C_16_H_18_O_9_	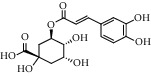

## Data Availability

All data used to support the findings of this study can be made available from the corresponding author upon request.

## Supplementary Materials

Supplementary Materials S1: 2D structures for the major bioactive constituents of *U. tomentosa*. S2: LC-MS data for spirooxindole alkaloids: speciophylline, isopteropodine, isomitraphylline, uncarine F, mitraphylline, and pteropodine. S3: LC-MS data for spirooxindole alkaloids: rhynchophylline and isorynchophylline. S4: LC-MS data for indole glycoside alkaloids: 3-dihydrocadambine. S5: LC-MS data for quinovic acid glycosides: QAG-1 and QAG-2. S6: LC-MS data for proanthocyanidins: proanthocyanidin C1, epiafzelechin-4*β*-8, proanthocyanidin B2/B4, epicatechin, and chlorogenic acid.
