# Supplementary material for: The Hydroalcoholic Extract of Uncaria tomentosa (Cat's Claw) Inhibits the Infection of Severe Acute Respiratory Syndrome Coronavirus 2 (SARS-CoV-2) In Vitro
Source: Evid Based Complement Alternat Med. 2021 Feb 24;2021:6679761. doi: 10.1155/2021/6679761 (PMC7929665; doi:10.1155/2021/6679761)
Supplement: Supplementary Materials — S1: 2D structures for the major bioactive constituents of U. tomentosa. S2: LC-MS data for spirooxindole alkaloids: speciophylline, isopteropodine, isomitraphylline, uncarine F, mitraphylline, and pteropodine. S3: LC-MS data for spirooxindole alkaloids: rhynchophylline and isorynchophylline. S4: LC-MS data for indole glycoside alkaloids: 3-dihydrocadambine. S5: LC-MS data for quinovic acid glycosides: QAG-1 and QAG-2. S6: LC-MS data for proanthocyanidins: proanthocyanidin C1, epiafzelechin-4β-8, proanthocyanidin B2/B4, epicatechin, and chlorogenic acid. [file 6679761.f1.doc]

| **LC-MS results**  2D-structures for the major bioactive constituents of the *U. tomentosa*  LC-MS data for Spiroxindole alkaloids: Speciophylline, Isopteropodine, Isomitraphylline, Uncarine F, Mitraphylline and Pteropodine.  LC-MS data for Spiroxindole alkaloids: Rynchophylline and Isorynchophyllin.  LC-MS data for Indole glycosides alkaloids: 3-dihydrocadambine  LC-MS data for Quinovic acid glycosides: QAG-1 and QAG-2  LC-MS data for Proanthocyanidins: Proanthocyanidin C1, Epiafzelechin-4β-8, Proanthocyanidin B2/B4, Epicatechin and Chlorogenic acid. | ***S1***  ***S2***  ***S3***  ***S4***  ***S5***  ***S6*** |
| --- | --- |

**Table of contents**

**S1. 2D-structures for the major bioactive constituents of the *U. tomentosa***

**S2. LC-MS data for Spiroxindole alkaloids: Speciophylline, Isopteropodine, Isomitraphylline, Uncarine F, Mitraphylline and Pteropodine**

**S3. LC-MS data for Spiroxindole alkaloids: Rynchophylline and Isorynchophyllin**

**S4. LC-MS data for Indole glycosides alkaloids: 3-dihydrocadambine**

**S5. LC-MS data for Quinovic acid glycosides: QAG-1 and QAG-2**

**S6.** **LC-MS data for Proanthocyanidins: Proanthocyanidin C1, Epiafzelechin-4β-8, Proanthocyanidin B2/B4, Epicatechin and Chlorogenic acid.**
